# LKB1 Regulates Inflammation of Fibroblast-like Synoviocytes from Patients with Rheumatoid Arthritis via AMPK-Dependent SLC7A11-NOX4-ROS Signaling

**DOI:** 10.3390/cells12091263

**Published:** 2023-04-26

**Authors:** Ha-Reum Lee, Su-Jin Yoo, Jinhyun Kim, Seong Wook Kang

**Affiliations:** 1 Research Institute for Medical Sciences, Chungnam National University School of Medicine, Daejeon 35015, Republic of Korea; hareum_lee@cnu.ac.kr (H.-R.L.); sujin428@cnuh.co.kr (S.-J.Y.); jkim@cnuh.co.kr (J.K.); 2 Division of Rheumatology, Department of Internal Medicine, Chungnam National University Hospital, Daejeon 35015, Republic of Korea

**Keywords:** rheumatoid arthritis, reactive oxygen species, fibroblast-like synoviocytes

## Abstract

Fibroblast-like synoviocytes (FLS) in rheumatoid arthritis (RA) patients have increased reactive oxygen species (ROS) levels and an impaired redox balance compared with FLS from control patients. Liver kinase B1 (LKB1) plays a key role in ROS scavenging and cellular metabolism in various cancers. Here, we aimed to determine the specific mechanism of LKB1 in RA pathogenesis. FLS were obtained from RA patients (n = 10). siRNA-induced LKB1 deficiency in RA FLS increased ROS levels via NADPH oxidase 4 (NOX4) upregulation. RA FLS migration and expression of inflammatory factors, including interleukin (IL)-1β, IL-6, IL-8, tumor necrosis factor-alpha (TNF-α), and vascular endothelial growth factor (VEGF), were enhanced by LKB1 deficiency. LKB1-deficient RA FLS showed increased sensitivity to oxidative stress damage caused by hydrogen peroxidase exposure. siRNA-induced solute carrier family 7 member 11 (SLC7A11) deficiency in RA FLS enhanced NOX4 and ROS expression and increased cell migration. When LKB1-deficient RA FLS were stimulated with an AMP-activated protein kinase (AMPK) activator, the LKB1-inhibition-induced cell migration significantly decreased through the restoration of SLC7A11/NOX4 expression. LKB1 regulates the AMPK-mediated SLC7A11-NOX4-ROS pathway to control cell migration and inflammation. Our data indicate that LKB1 is a key regulator of redox homeostasis in RA FLS.

## 1. Introduction

Rheumatoid arthritis (RA) is a systemic chronic autoimmune disease, which is characterized by inflamed joints and hyperplastic synovium that results in bone destruction [1,2]. Fibroblast-like synoviocytes (FLS) compose the intimal lining of the synovium and contribute to RA pathogenesis and progression [3]. RA FLS can migrate to and invade the synovium surrounding joint tissue and secrete diverse pro-inflammatory cytokines and extracellular protease enzymes [4]. During these activated responses, numerous immune cells are recruited into the synovium and stimulate excessive immune responses via direct communication with FLS [5]. Thus, aggressive FLS are major effectors in the development of the RA inflammatory microenvironment and are considered key targets in RA treatment.

Reactive oxygen species (ROS) are intracellular signaling molecules that function in the regulation of cell migration, chemotaxis, apoptosis, and inflammation [6]. Moderate concentrations of ROS are necessary for maintaining immune system homeostasis and protecting against inflammation, cancer, and autoimmune diseases [7]. However, RA patients show abnormally high ROS levels and glycolytic rates compared with healthy controls [8,9]. In a previous study, we reported that NADPH oxidase 4 (NOX4), a major ROS source, is upregulated in RA FLS following tumor necrosis factor-alpha (TNF-α) and interleukin (IL)-17 stimulation. Subsequently, aggressive RA FLS migration via the ROS/vascular cell adhesion molecule 1 (VCAM1)/vascular endothelial growth factor (VEGF) pathway occurred [10]. Furthermore, circulating T cells from RA patients showed increased ROS generation according to disease activity, and FLS inflammatory responses were suppressed via ROS inhibition in peripheral blood mononuclear cells [11]. Though ROS play a critical role in the interactions between FLS and immune cells and enhance RA severity [12], the specific mechanism of ROS-mediated cell migration remains unclear in RA FLS.

Liver kinase B1 (LKB1, also known as serine/threonine kinase 11, STK11) is a tumor suppressor gene and is involved in cellular apoptosis, metabolism, and oxidative responses [13,14]. Because LKB1 directly activates AMP-activated protein kinase (AMPK), a central metabolic sensor in glucose metabolism, energy homeostasis, and the oxidative phosphorylation system, LKB1 is considered a multifunctional master kinase [15,16]. However, the specific function of LKB1 in RA has not been examined.

In this study, we induced LKB1 deficiency in primary FLS from RA patients using siRNA with Lipofectamine transfection. LKB1 deficiency induced upregulation of intracellular mitochondrial-specific ROS levels and enhanced migration capacity through SLC7A11-NOX4 signaling. These effects were negated by the AMPK-specific activator A769662. Thus, LKB1 deficiency induces SLC7A11-NOX4-ROS expression that eventually leads to increased cell migration, thereby further exacerbating inflammation by RA FLS.

## 2. Materials and Methods

### 2.1. Human Subjects and Ethics Statement

Synovial tissues were obtained from RA patients (n = 10) and osteoarthritis (OA) patients (n = 10) who were undergoing synovectomy or joint replacement (Table 1). The diagnosis of RA conformed to the College of Rheumatology (ACR)/European League Against Rheumatism (EULAR) 2010 classification criteria [17]. The classification of OA was in accordance with the American College of Rheumatology (ACR) criteria [18]. Patients were excluded if any of the following disorders were present: inflammatory diseases, such as septic arthritis, history of knee surgery, infectious diseases, cancer, multiple sclerosis, stroke, or immune system disorder. After removing fat and fibrous tissues, the synovium was cut into small pieces and incubated with 0.1% collagenase (Sigma-Aldrich, St. Louis, MO, USA) in Dulbecco’s Modified Eagle’s Medium (DMEM, Gibco, Thermo Fisher Scientific, Waltham, MA, USA) at 37 °C for 2 h. The purity of the isolated primary FLS (CD90^+^) was greater than 95% and used for experiments after four to six passages. Cells were cultured in DMEM supplemented with 10% fetal bovine serum (FBS, Gibco, Thermo Fisher Scientific) and maintained in a 5% CO_2_ incubator at 37 °C. This study was performed according to the recommendations of the Declaration of Helsinki and was approved by the Institutional Review Board of Chungnam National University Hospital (CNUH 2019-12-068). A written informed consent was obtained from each patient.

### 2.2. Reverse Transcription (RT)-PCR and Quantitative (q)RT-PCR

Total RNA was extracted from FLS using TRI Reagent (Molecular Research Center, Cincinnati, OH, USA) according to the manufacturer’s instructions. Extracted RNA was reverse transcribed with ReverTra Ace^®^ qPCR RT Master Mix (TOYOBO, Osaka, Japan) according to the manufacturer’s instructions. SYBR^®^ Green Real-Time PCR Master Mix (TOYOBO) was used for qRT-PCR analysis of cDNA according to the manufacturer’s instructions. Thermal cycling was conducted on a CFX Connect Real-Time PCR Detection System machine (Bio-Rad Laboratories, Hercules, CA, USA). Target gene expression levels are shown as a ratio of the level of glyceraldehyde 3-phosphate dehydrogenase (GAPDH) in the same sample via calculation of the cycle threshold (Ct) value. Relative expression levels of target genes were calculated using the 2^−ΔΔCT^ relative quantification method.

For RT-PCR, the synthesized cDNA was mixed with AccuPower^®^ RT PreMix (Bioneer, Daejeon, Republic of Korea; see Table 2 for primer sequences) and specific PCR primers following the manufacturer’s protocol. Amplified products were separated on 1% agarose gels, stained with Midori green advance (NIPPON Genetics, Düren, Germany), and photographed under UV illumination using a GelDoc system (Bio-Rad Laboratories).

### 2.3. Western Blot Analysis

FLS were lysed on ice using a RIPA lysis kit (ATTO, Co., Tokyo, Japan; containing protease and phosphatase inhibitors). Lysates were clarified by centrifugation, and samples were analyzed by sodium dodecyl sulfate–polyacrylamide gel electrophoresis (SDS-PAGE). Proteins were transferred onto polyvinylidene fluoride (PVDF) membranes (Bio-Rad Laboratories), which were then incubated with antibodies against phospho-LKB1 (Ser428), LKB1, p-NF-κB p65 (Ser536), NF-κB p65, phospho-p38 MAPK (Thr180/Tyr182), p38-MAPK, phospho-AMPK (Thr172), AMPK, SLC7A11, GAPDH (all obtained from Cell Signaling Technology, Danvers, MA, USA), or NOX4 (Abcam, Cambridge, UK) overnight at 4 °C. After washing with PBS-Tween 20, membranes were stained with peroxidase-conjugated goat anti-rabbit IgG (AbFrontier Co., Seoul, Republic of Korea). Target proteins were detected using a chemiluminescent HRP Substrate (Thermo Fisher Scientific).

### 2.4. siRNA Transfection

Specific siRNAs targeting human LKB1 or SLC7A11 were purchased from Santa Cruz Biotechnology (Dallas, TX, USA). Scramble siRNA (Santa Cruz Biotechnology) was used as the control. FLS were transfected with Lipofectamine 3000 transfection reagent (Invitrogen, Carlsbad, CA, USA) and the indicated siRNA constructs. After incubation for 24 h, target gene expression was evaluated by RT-PCR or western blot analysis.

### 2.5. Flow Cytometric Analysis

To detect ROS levels, FLS were stained with CellROX or MitoSOX™ Red mitochondrial superoxide indicator (Invitrogen, Thermo Fisher Scientific) according to the manufacturer’s instructions. To determine mitochondrial membrane potentials, TMRM (Tetramethylrhodamine, Methyl Ester, Perchlorate; Invitrogen, Thermo Fisher Scientific) was used according to the manufacturer’s instructions. FLS were analyzed on a FACSCanto2 flow cytometer (BD Biosciences, Franklin Lakes, NJ, USA), and data were processed using FlowJo software v 10.8 (Tree Star, Ashland, OR, USA).

GLX351322 (MedKoo Biosciences, Morrisville, NC, USA) was used to inhibit NOX4 in these cellular processes, and MitoTEMPO (Sigma-Aldrich) was used as a specific scavenger of mitochondrial superoxide. Human recombinant TNF-a (10 ng/mL) and IL-17 (10 ng/mL) were obtained from PeproTech (Cranbury, NJ, USA). A769662 (Sigma-Aldrich) was employed for activating AMPK-mediated cellular signaling.

### 2.6. Enzyme-Linked Immunosorbent Assay (ELISA)

IL-6, IL-8, TNF-α, and VEGF concentrations were measured using ELISA kits for human IL-6 (BD Biosciences), IL-8 (R&D Systems, Minneapolis, MN, USA), TNF-α (BD Biosciences), and human VEGF (R&D Systems) according to the manufacturers’ instructions. Protein levels were estimated by interpolation from a standard curve generated using a Sunrise absorbance reader (Tecan, Männedorf, Switzerland) at 450 nm.

### 2.7. Transwell Migration Assay

Following transfection with LKB1 siRNA for 24 h, RA FLS were centrifuged and loaded onto Transwell filters with 8 mm pores (Corning, Inc., Corning, NY, USA) positioned on top of migration chambers for 24 h. DMEM containing 10% FBS was placed in the bottom chamber of the Transwell plate as a chemoattractant. Non-migrating cells on the top membrane surface were removed by washing with PBS and cotton swabs. Migrated cells were stained with crystal violet dye and counted in five random fields per sample under an inverted microscope (Olympus, Tokyo, Japan; magnification, 100×; 0.55 numerical aperture dry objective; Scale bar, 100 mm).

### 2.8. Statistical Analysis

Results are presented as mean ± standard deviation (SD). The level of significance, determined at the 95% confidence limit or greater (*p* < 0.05), was calculated using one-way analysis of variance (ANOVA), followed by Duncan’s post hoc tests using SPSS 22.0 (IBM, Armonk, NY, USA). Different letters (a, b, c, d, and e) indicate statistically significant differences among groups (*p* < 0.05). Additional statistical analyses were performed using paired Student’s *t*-tests, with *p* < 0.05 considered statistically significant.

## 3. Results

### 3.1. LKB1 Knockdown Increased ROS Levels via Increased NOX4 Expression in RA FLS

**Figure 1:**
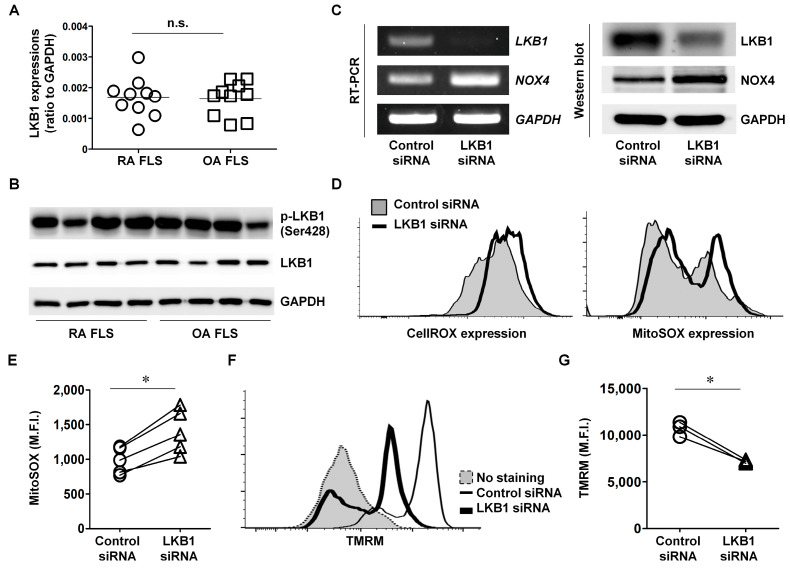
LKB1 deficiency results in increased mitochondrial-specific ROS levels in RA FLS. (**A**,**B**) LKB1 expression in RA FLS (n = 10) and OA FLS (n = 10) was analyzed by qRT-PCR and western blot. GAPDH was used as a loading control for western blot analysis. (**C**) RA FLS were transfected with LKB1 or control siRNA. After 24 h, LKB1 and NOX4 mRNA and protein expression levels were analyzed by RT-PCR and western blot, respectively. Data are from a representative experiment that was performed in triplicate. GAPDH was used as the loading control. (**D**) Intracellular ROS levels were detected by staining RA FLS transfected with LKB1 or control siRNA with CellROX or MitoSOX. (**E**) Data represent the mean fluorescence intensity (M.F.I.) for MitoSOX. (**F**) RA FLS were transfected with LKB1 or control siRNA. After 24 h, mitochondrial membrane potential was analyzed using TMRM staining. (**G**) Data represent the mean fluorescence intensity (M.F.I.) for TMRM. Each symbol represents an individual donor. Statistical analysis was performed by one-way ANOVA, followed by Duncan’s post hoc test. * *p* < 0.05. n.s.: not significant.

LKB1 plays a key role in redox homeostasis and ROS scavenging in cancer [19]. Therefore, we examined constitutive LKB1 levels in FLS from RA and OA patients. LKB1 mRNA and phosphorylated protein levels showed no differences between RA and OA FLS in this study (n = 10 per group; Figure 1A,B).

To determine whether LKB1 regulates intracellular ROS levels in RA, RA FLS were transfected with LKB1 siRNA. After 24 h, both LBK1 mRNA and protein levels were reduced (Figure 1C). NOX4 expression was markedly elevated in LKB1-deficient RA FLS compared with control siRNA-transfected RA FLS. Because NOX4 is an enzyme involved in ROS generation, intracellular ROS levels were measured in LKB1-deficient FLS [20]. LKB1-deficient RA FLS had significantly increased mitochondrial and intracellular ROS levels compared with control siRNA-transfected RA FLS (n = 5; * *p* < 0.05; Figure 1D,E). Mitochondrial potentials were also decreased by LKB1 inhibition in RA FLS compared with control RA FLS (n = 5; * *p* < 0.05; Figure 1F,G). These data indicate that LKB1 regulates ROS expression in RA FLS via enhanced NOX4 expression.

### 3.2. LKB1 Regulated Inflammatory Cytokine Production and Cell Migration in RA FLS

**Figure 2:**
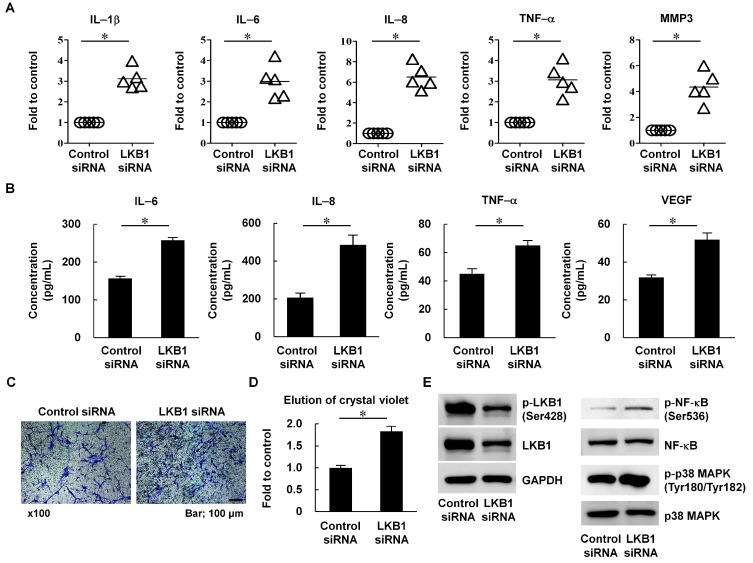
LKB1 deficiency induces inflammation and migration in RA FLS. (**A**) RA FLS were transfected with LKB1 or control siRNA. Cytokine mRNA levels were assessed by qRT-PCR (n = 5). GAPDH was used as a control. Data are presented as fold change compared with GAPDH. Each symbol represents an individual donor. (**B**) Secreted levels of IL-6, IL-8, TNF-α, and VEGF in culture supernatants of RA FLS transfected with LKB1 or control siRNA were measured by ELISAs (n = 5). Results are expressed as mean ± SD. (**C**,**D**) Cell migration by RA FLS transfected with LKB1 or control siRNA was measured using Transwell chambers for 24 h. (**C**) Representative Transwell chambers with crystal violet stained migrated cells are shown. Scale bar, 100 mm. Magnification was 100×. (**D**) After solubilization, crystal violet dye intensity was measured (n = 5). Data represent the fold change in optical density of crystal violet stained cells compared with control siRNA. Results are expressed as means ± SD. (**E**) RA FLS were transfected with LKB1 or control siRNA. After 24 h, cell lysates were separated by SDS-PAGE and analyzed by western blot analysis with specific target antibodies. GAPDH was used as the control. Data are from a representative experiment that was performed in triplicate. Statistical analysis was performed using one-way ANOVA, followed by Duncan’s post hoc test. * *p* < 0.05.

ROS are related to pro-inflammatory cytokine production [21]. Therefore, we investigated whether LKB1 modulates the expression of cytokines known to cause RA aggravation, including IL-1 β, TNF-a, and matrix metalloproteinase 3 (MMP3) [22]. Following LKB1 knockdown in RA FLS, both mRNA and culture supernatant protein levels were examined. IL-1β, IL-6, IL-8, TNF-a, and MMP3 mRNA and protein levels were significantly increased in LKB1-deficient RA FLS compared with control siRNA-transfected RA FLS (n = 5 per group; * *p* < 0.05; Figure 2A,B).

Increased ROS and cytokine levels can induce FLS migration [10]. Therefore, Transwell migration assays were performed. Inhibition of LKB1 increased migration by RA FLS compared with control siRNA-transfected RA FLS (n = 5; 1.83-fold; * *p* < 0.05; Figure 2C,D). When RA FLS were transfected with LKB1 siRNA, phosphorylation of NF-κB and p38 MAPK was increased compared with RA FLS transfected with control siRNA (Figure 2E). These findings suggest that LKB1 expression negatively regulates pro-inflammatory signaling and cytokine production in RA FLS.

### 3.3. LKB1-Deficient RA FLS Were Highly Sensitive to ROS-Mediated Inflammation

**Figure 3:**
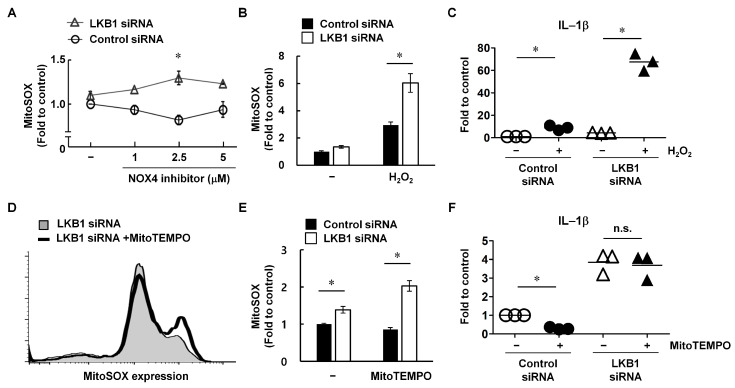
LKB1-deficient RA FLS lack a protective effect against H_2_O_2_-induced damage. (**A**) RA FLS were transfected with LKB1 or control siRNA for 24 h and then treated with GLX351322, a NOX4 inhibitor. Following incubation for 3 h, intracellular ROS levels were measured using MitoSOX. Data are presented as fold change compared with untreated cells. Data are from a representative experiment that was performed in triplicate. Results are expressed as mean ± SD. Statistical analysis between LKB1-deficient and control RA FLS treated with GLX351322 (2.5 mM) was performed using the paired Student’s *t*-test. * *p* < 0.05. (**B**) RA FLS were transfected with LKB1 or control siRNA for 24 h and then treated with H_2_O_2_ (1 mM) for 3 h. ROS levels were analyzed using MitoSOX staining (n = 3). Data represent the mean fluorescence intensity (M.F.I.) for MitoSOX. Results are expressed as mean ± SD. (**C**) RA FLS were transfected with LKB1 or control siRNA for 24 h and then treated with H_2_O_2_ (1 mM) for 3 h. IL-1β mRNA levels were analyzed using qRT-PCR (n = 3). Data are presented as fold change compared with GAPDH. (**D**,**E**) RA FLS were transfected with LKB1 or control siRNA for 24 h and then treated with MitoTEMPO (10 mM) for 1 h. (**D**) ROS levels were analyzed using MitoSOX staining. (**E**) Data represent the mean fluorescence intensity (M.F.I.) for MitoSOX (n = 3). Results are expressed as mean ± SD. (**F**) RA FLS were transfected with LKB1 or control siRNA for 24 h and then treated with MitoTEMPO (10 mM) for 1 h. IL-1β mRNA levels were analyzed using qRT-PCR (n = 3). Data are presented as fold change compared with GAPDH. Statistical analysis was performed using one-way ANOVA, followed by Duncan’s post hoc test. * *p* < 0.05. n.s.: not significant.

To determine whether a NOX4 inhibitor could recover LKB1-mediated signaling in LKB1-deficient RA FLS, RA FLS were transfected with LKB1 siRNA and then incubated with a NOX4 inhibitor (GLX351322). In RA FLS transfected with control siRNA and treated with 2.5 mM NOX4 inhibitor, mitochondrial ROS levels were reduced by 18.4% compared with similar cells not treated with NOX4 inhibitor (* *p* < 0.05; Figure 3A). However, mitochondrial ROS levels in LKB1-deficient RA FLS increased by 29.8% when treated with 2.5 mM NOX4 inhibitor compared with similar cells without NOX4 inhibitor. Therefore, LKB1 knockdown may induce irreversible oxidative stress damage in RA FLS.

Exogenous H_2_O_2_ is a form of ROS that can lead to mitochondrial dysfunction, expression of inflammatory factors, and cell death [23]. Furthermore, H_2_O_2_ is the primary product (approximately 90%) of NOX4 activity [24], and NOX4-dependent ROS generation is required for IL-1β expression [25]. Therefore, we transfected RA FLS with LKB1 siRNA and then exposed them to H_2_O_2_. Exposure of LKB1-deficient RA FLS to 1 mM H_2_O_2_ for 3 h significantly enhanced ROS generation compared with RA FLS transfected with control siRNA (* *p* < 0.05; Figure 3B). IL-1β expression in LKB1-deficient RA FLS was also significantly increased by H_2_O_2_ treatment compared with control siRNA-transfected RA FLS (* *p* < 0.05; Figure 3C). ROS expression was reduced in control siRNA-transfected RA FLS following treatment with MitoTEMPO, a specific scavenger of mitochondrial superoxide (* *p* < 0.05; Figure 3D,E). However, MitoTEMPO-treated LKB1-deficient RA FLS had increased ROS levels. MitoTEMPO also inhibited IL-1β expression in control cells, whereas LKB1-deficient RA FLS showed no alteration in IL-1β expression (* *p* < 0.05; Figure 3F). Therefore, LKB1 may regulate the ROS balance and protect against ROS-mediated damage in RA FLS.

### 3.4. SLC7A11 Regulated LKB1-Mediated Cell Migration through NOX4 Signaling

**Figure 4:**
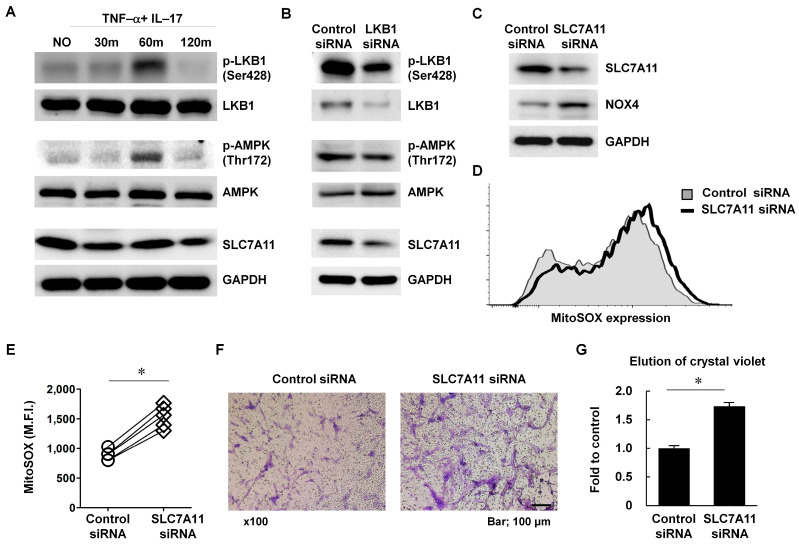
LKB1-mediated signaling occurred through regulation of the SLC7A11-NOX4 pathway in RA FLS. (**A**) RA FLS were stimulated with TNF-α (10 ng/mL) and IL-17 (10 ng/mL) for 30, 60, or 120 min. Cell lysates were assessed for LKB1 and AMPK phosphorylation and SLC7A11 levels by western blot analysis. (**B**) After RA FLS were transfected with LKB1 or control siRNA for 24 h, the expression levels of LKB1, APMK, and SLC7A11 were analyzed. (**C**) After RA FLS were transfected with SLC7A11 or control siRNA for 24 h, protein levels of SLC7A11 and NOX4 were examined. Data are from a representative experiment that was performed in triplicate. (**D**,**E**) After RA FLS were transfected with SLC7A11 or control siRNA for 24 h, (**D**) intracellular ROS levels were detected by MitoSOX. (**E**) Data are presented as the mean fluorescence intensity (M.F.I.) for MitoSOX. Each symbol represents an individual donor (n = 5). (**F**,**G**) Cell migration by RA FLS transfected with SLC7A11 or control siRNA was measured using Transwell chambers for 24 h. (**F**) Representative Transwell chambers with crystal violet stained migrated cells are shown. Scale bar, 100 mm. Magnification was 100×. (**G**) After solubilization, crystal violet dye intensity was measured (n = 5). Data represent the fold change in optical density of crystal violet stained cells compared with the control siRNA. Results are expressed as mean ± SD. Statistical analysis was performed using one-way ANOVA, followed by Duncan’s post hoc test. * *p* < 0.05.

There is a close correspondence between solute carrier family 7 member 11 (SLC7A11; also known as xCT) and AMPK, which is directly phosphorylated by LKB1 [26,27]. To determine the role of SLC7A11 in LKB1-mediated signaling, RA FLS were stimulated with recombinant human TNF-α and IL-17, as they induce pro-inflammatory conditions in RA FLS [28]. Following treatment with TNF-α and IL-17 for 1 h, LKB1 and AMPK phosphorylation were upregulated in RA FLS (Figure 4A). However, SLC7A11 expression gradually decreased following cytokine treatment. When LKB1 expression was downregulated in RA FLS, SLC7A11 levels also decreased (Figure 4B).

To determine more precisely the function of SLC7A11 in the regulation of mitochondrial-specific ROS production, RA FLS were transfected with SLC7A11 siRNA. Following SLC7A11 inhibition, NOX4 expression was markedly increased and ROS levels were significantly upregulated compared with control siRNA-transfected RA FLS (n = 5; Figure 4C–E). Cell migration was also enhanced in SLC7A11-deficient RA FLS (n = 5, * *p* < 0.05; Figure 4F,G). Based on these results, LKB1 may regulate the SLC7A11-NOX4-ROS pathway in RA FLS.

### 3.5. The Effects of LKB1 Deficiency Were Overcome in RA FLS by Treatment with an AMPK Activator

**Figure 5:**
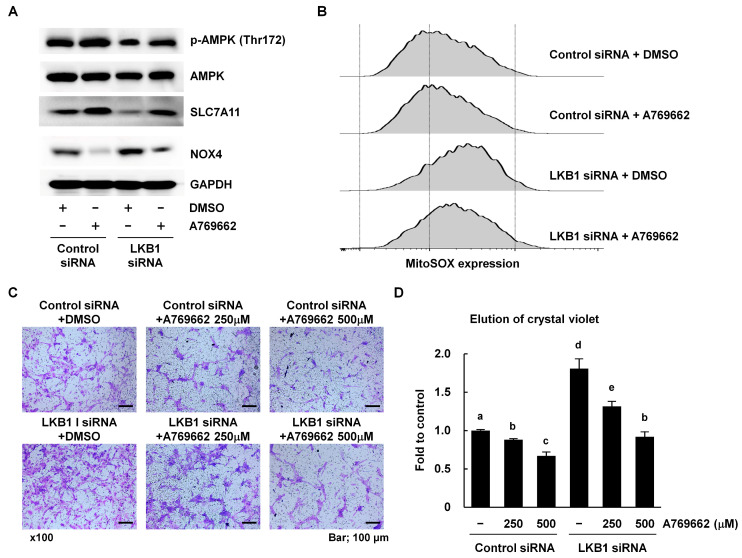
The AMPK activator A769962 restored LKB1-deficient RA FLS. (**A**) RA FLS were transfected with LKB1 or control siRNA for 24 h and then stimulated with A769962 (500 mM) for 2 h. Western blots were used to measure AMPK, SLC7A11, and NOX4 protein in cell lysates. DMSO was used as the solvent control. Data are from a representative experiment that was performed in triplicate. (**B**) RA FLS were transfected with LKB1 or control siRNA for 24 h and then incubated with A769962 (500 mM) for 16 h. Intracellular ROS levels were analyzed using MitoSOX staining. Data are from a representative experiment that was performed in triplicate. (**C**,**D**) RA FLS were transfected with LKB1 or control siRNA for 24 h and then treated with A769962 (250 or 500 mM) for 2 h. Cell migration was measured using Transwell chambers for 24 h. (**C**) Representative Transwell chambers with crystal violet stained migrated cells are shown. Scale bar, 100 mm. Magnification was 100×. (**D**) After solubilization, crystal violet dye intensity was measured (n = 5). Data represent the fold change in optical density of crystal violet stained cells compared with the control siRNA. Results are expressed as means ± SD. Statistical analysis was performed using one-way ANOVA, followed by Duncan’s post hoc test. Different letters (a, b, c, d, and e) indicate statistically significant differences (*p* < 0.05).

To determine whether AMPK activation could alleviate the effects of LKB1 deficiency, LKB1-deficient RA FLS were treated with A769662 which stimulates AMPK Thr172 phosphorylation and its downstream signaling [29]. Although LKB1 remained inhibited, treatment with the AMPK activator recovered SLC7A11 and NOX4 expression in RA FLS (Figure 5A). Furthermore, ROS levels and cell migration were downregulated following treatment with the AMPK activator in LKB1-deficient RA FLS (Figure 5B–D). Taken together, these results suggest that LKB1-mediated AMPK signaling modulates the SLC7A11-NOX4-ROS pathway, ultimately leading to cell migration regulation. Therefore, LKB1 is a key regulator of redox homeostasis in RA FLS.

## 4. Discussion

LKB1 plays an essential role in cell metabolism and oxidative stress within the tumor microenvironment [30]. In a lipopolysaccharide (LPS)-induced lung injury model, LKB1 prevents inflammatory responses and ROS production via the AMPK/NLR family pyrin-domain-containing 3 (NLRP3) pathway [31]. Additionally, LKB1 regulates angiogenesis and invasion of breast cancer by suppressing expression of VEGF and MMPs [32]. Although there are many studies on LKB1 in cancer settings, the role of LKB1 has not been reported in relation to RA.

When LKB1 expression was suppressed via siRNA, RA FLS showed increased NOX4 and ROS levels as well as increased damage to the mitochondrial membrane potential (Figure 1C–G). These changes resulted in increased inflammation and migration capacity in RA FLS (Figure 2). LKB1 regulates mitochondrial functions and dynamics and ROS levels through AMPK-related downstream kinases [33]. Because mitochondria perform many key functions through oxidative phosphorylation and act as regulators of cellular metabolism, LKB1-mediated signaling is considered a master regulator of several metabolic pathways involved in RA pathogenesis [34]. Moreover, LKB1 overexpression stimulates antioxidant pathways, resulting in increased resistance to oxidative stress through NRF2 phosphorylation [35]. In this study, LKB1-deficient RA FLS were highly sensitive to oxidative stress, which may result in irreversible damage (Figure 3). LKB1 deficiency induces bone marrow failure and regulates hematopoietic stem cell survival [36]. Additionally, endothelial-cell-specific LKB1-deficient mice show tumor development, weight loss, and natural death [37]. Therefore, LKB1 may be involved in cell homeostasis and survival against oxidative stress and metabolic imbalance in RA.

SLC7A11 is a multifunctional protein that functions in cellular redox homeostasis and tumor malignancy through the activation of the antioxidant glutathione (GSH) [38]. LKB1 inhibition in RA FLS resulted in reduced SLC7A11 expression, and SLC7A11 deficiency resulted in increased ROS levels and enhanced migratory capacity in RA FLS (Figure 4). We first reported that LKB1 modulates intracellular ROS levels through SLC7A11 expression in RA as excessive ROS stimulate cell migration, and cell death is also promoted by oxidative stress [39]. Furthermore, SLC7A11 inactivation is related to ferroptosis (an iron-dependent, nonapoptotic cell death triggered by an impaired antioxidant system) via AMPK signaling; thus, SLC7A11 may be a new potential target for overcoming cancer resistance [40,41]. Further studies are needed to determine whether LKB1-mediated SLC7A11 signaling is related to ferroptosis in RA FLS.

Metformin-activated AMPK expression reduces cartilage degradation and limits OA development in an injury-induced mouse model [42]. Administration of metformin in RA patients shows anti-inflammatory effects, reduced disease severity, and improved quality of life compared with the control group [43]. However, the specific mechanism of metformin is unknown. In this study, the AMPK activator A769662 restored LKB1-loss-induced cell migration through SLC7A11-NOX4 upregulation (Figure 5). On the other hand, AMPK is involved in modulating the TH17-Treg imbalance via STAT3 inhibition in a collagen-antibody-induced arthritis model [44]. If LKB1-mediated signaling regulates both FLS inflammation and the hyperactivated immune response, LKB1 could be a key treatment target in RA. Furthermore, these findings could extend its therapeutic effect to other autoimmune and inflammation-related diseases.

Limitations of this study include that it utilized a small number of FLS from RA patients and that the in vitro findings may not precisely reflect in vivo responses. Therefore, expanding this study to an animal model of RA would be beneficial. Collectively, these results suggest that LKB1-mediated AMPK signaling modulates the SLC7A11-NOX4-ROS pathway, ultimately leading to regulation of cell migration in FLS from patients with RA. We first reported that LKB1 modulates intracellular ROS production through SLC7A11 expression in RA as excessive ROS stimulate cell migration, and cell death is also promoted by oxidative stress. Although it was reported that leptin induces LKB1-AMPK-mediated inflammation of RA FLS [45], the LKB1-AMPK-SLC7A11-NOX4-ROS pathway has never been reported. Moreover, LKB1-deficient RA FLS were highly sensitive to oxidative stress, which may result in irreversible damage. These imply that LKB1-regulated SLC7A11 signaling is a novel pathway in RA inflammation, thus contributing to a better understanding of RA pathogenesis.

## 5. Conclusions

LKB1-mediated AMPK signaling modulates the SLC7A11-NOX4-ROS pathway, ultimately leading to cell migration regulation in RA FLS. Our data indicate that LKB1 is a key regulator of redox homeostasis in RA FLS.

## Figures and Tables

**Table 1:** Baseline characteristics of all patients.

Variable		Subjects with Knee RA (n = 10)	Subjects with Knee OA (n = 10)
Female (n, %)		7 (70)	7 (70)
Age (year, mean ± SD)		55.2 ± 9.3 (35–62)	68.2 ± 9.8 (50-84)
Duration of disease (month, mean ± SD)		130.6 ± 88.7 (40–348)	NA
Rheumatoid factor–positive, n (%)		9/9 (100)	NA
Anti-CCP antibody–positive, n (%)		5/7 (71.4)	NA
DAS28 (ESR, mean ± SD)		2.86 ± 1.31 (1.64–5.81)	NA
Duration of treatment (month, mean ± SD)		118.5 ± 94.2 (28–336)	NA
Treatment (n, %)	Naïve	0	NA
	Steroid	10 (100)	
	Methotrexate	8 (80)	
	Hydroxychloroquine	5 (50)	
	Sulfasalazine	3 (30)	
	Leflunomide	6 (60)	
	Tacrolimus	1 (10)	
	Biologic DMARD	1 (10), Golimumab	
Kellgren–Lawrence score (grade, mean ± SD)		NA	3.3 ± 0.48 (3–4)

SD: standard deviation; NA: not applicable; CCP: cyclic citrullinated peptide; DAS28: disease activity score of 28 joint counts; DMARD: disease-modifying antirheumatic drug.

**Table 2:** Primers used for PCR.

	Sense Primer	Antisense Primer
*LKB1*	CTGAGTACGAACCGGCCAA	CTACGGCACCACAGTCATG
*NOX4*	CTCAGCGGAATCAATCAGCTGTG	AGAGGAACACGACAATCAGCCTTAG
*IL-1β*	GGATATGGAGCAACAAGTGG	ATGTACCAGTTGGGGAACTG
*IL-6*	AACCTGAACCTTCCAAAGATGG	TCTGGCTTGTTCCTCACTACT
*IL-8*	CATACTCCAAACCTTTCCACCCC	TCAGCCCTCTTCAAAAACTTCTCCA
*TNF-α*	CCCGAGTGACAAGCCTGTAG	GATGGCAGAGAGGAGGTTGAC
MMP-3	GATGCCCACTTTGATGATGATGAA	AGTGTTGGCTGAGTGAAAGAGACC
*GAPDH*	CACATGGCCTCCAAGGAGTAA	TGAGGGTCTCTCTCTTCCTCTTGT

## Data Availability

Not applicable.
